# Patient with Marfan Syndrome and a Novel Variant in FBN1 Presenting with Bilateral Popliteal Artery Aneurysm

**DOI:** 10.1155/2018/6780494

**Published:** 2018-03-29

**Authors:** Ahmed Mohammad, Haytham Helmi, Paldeep S. Atwal

**Affiliations:** ^1^Department of Clinical Genomics, Mayo Clinic, Jacksonville, FL, USA; ^2^Center for Individualized Medicine, Mayo Clinic, Jacksonville, FL, USA

## Abstract

We present a 43-year-old man with aortic root dilation, mitral valve prolapse, and marfanoid appearance, who presented with acute onset left leg pain. He underwent a Doppler ultrasound that revealed left popliteal artery aneurysm with thrombus. CT angiogram showed bilateral popliteal artery aneurysms. After repairing of his left popliteal artery aneurysm, he was sent for genetic evaluation. He was diagnosed with Marfan syndrome (MFS) based on the revised Ghent criteria and then underwent* FBN1* sequencing and deletion/duplication analysis, which detected a novel pathogenic variant in gene* FBN1*, denoted by c.5872 T>A (p.Cys1958Ser). MFS is a connective tissue disorder with an autosomal dominant inheritance due to pathogenic variants in* FBN1* that encodes Fibrillin-1, a major element of the extracellular matrix, and connective tissue throughout the body. MFS involves multiple systems, most commonly the cardiovascular, musculoskeletal, and visual systems. In our case we present a rare finding of bilateral popliteal artery aneurysms in a male patient with MFS.

## 1. Background

MFS is a genetic connective tissue disorder with an autosomal dominant inheritance that involves multiple systems, most commonly the cardiovascular, musculoskeletal, and ocular systems [1]. It is caused by pathogenic variants in* FBN1* that encodes Fibrillin-1, a major element of the extracellular matrix, and connective tissue throughout the body [2]. Due to the improved survival and longer life expectancy of patients with MFS, less common phenotypic features continue to be observed [3]. MFS is diagnosed based on Ghent criteria, which takes into account clinical manifestations, genetic testing, and family history [4]. In this report we present a male patient with clinical features of MFS, a novel pathogenic variant in* FBN1,* and the unusual finding of bilateral popliteal aneurysms.

## 2. Case Presentation

**Figure 1:**
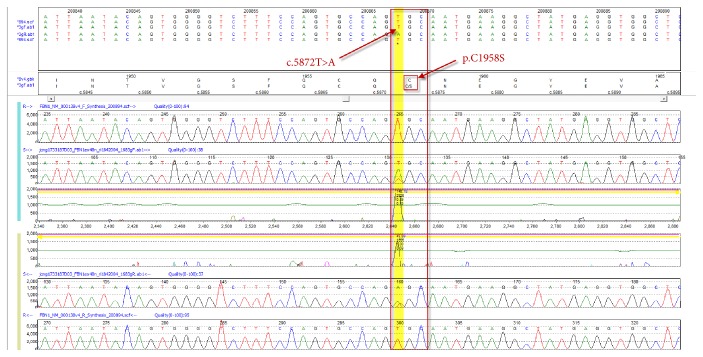
DNA chromatogram demonstrating heterozygous c.5872T>A variant in* FBN1*.

**Figure 2:**
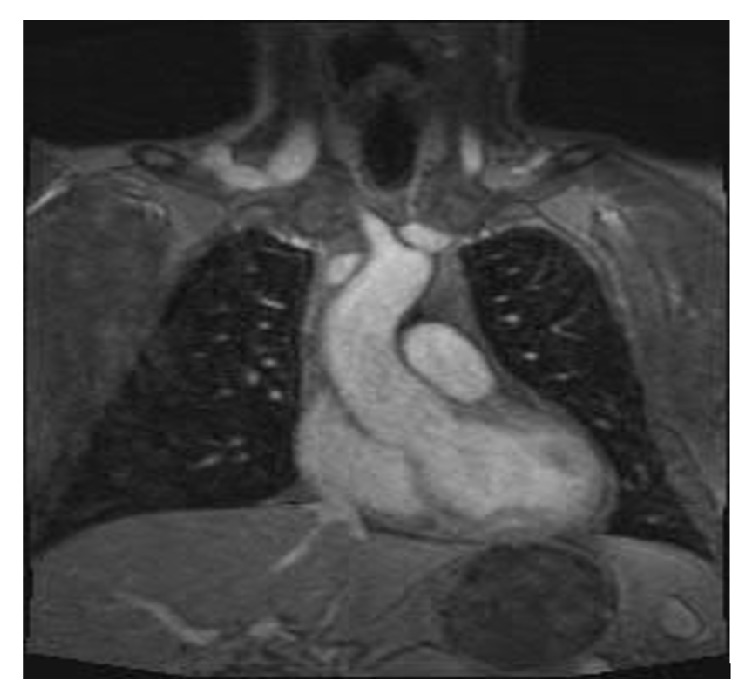
MRI of the heart with and without intravenous gadolinium contrast administration.

**Figure 3:**
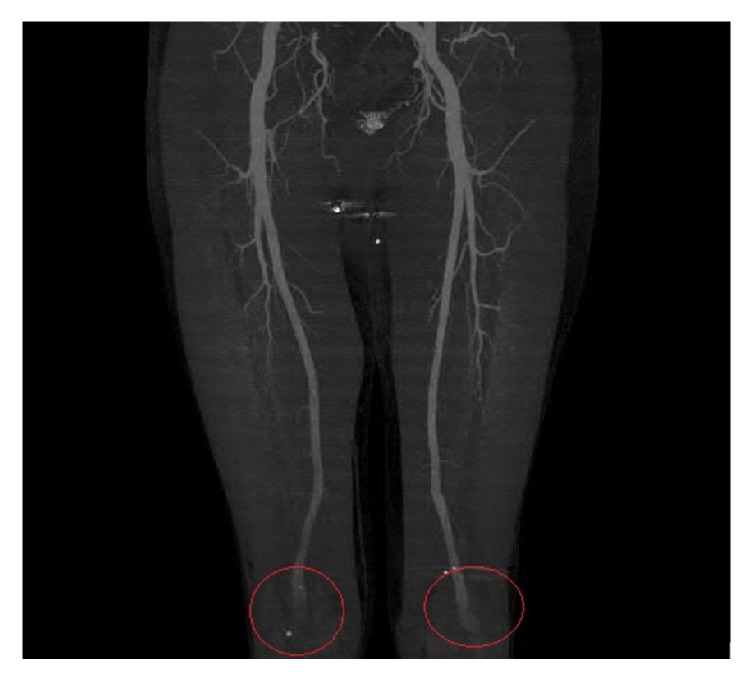
CT angiogram MIP images showing bilateral popliteal aneurysms highlighted by red circles.

Our proband is a 43-year-old male of northern European descent who initially presented with acute onset left leg cramping pain that lasted for several days. The pain was associated with numbness in his left leg and nausea. His past medical history was significant for mitral valve prolapse, aortic root dilation umbilical hernia left anterior cruciate ligament (ACL) rupture, and severe myopia corrected with LASIK surgery. Family history revealed rheumatic heart disease in his mother, necessitating mitral valve repair due to mitral valve prolapse with severe regurgitation. Upon examination, his popliteal arterial pulses were 4+ bilateral, bounding. His height was 196 cm, with an increased arm span-height ratio of 1.07. He had a mild pectus carinatum deformity, mild scoliosis positive wrist and thumb sign, pes planus, and mild skin striae. A Doppler ultrasound was performed by his primary care physician (PCP), which ruled out deep venous thrombosis (DVT); however, it revealed a large left popliteal artery aneurysm with thrombus that measured 6.3 × 3.1 × 3.4 cm. He underwent a CT angiogram that showed normal abdominal aorta as well as common iliac arteries. However it showed bilateral popliteal artery aneurysms measuring 3.6 × 4 cm in the left side and 3 × 2.8 cm in the right side (Figure 3). After repairing of his left popliteal artery aneurysm with bovine carotid interposition via posterior approach, he was sent for cardiac and genetic evaluations. He underwent a MRI exam of the heart with and without intravenous gadolinium contrast administration (Figure 2) which showed mild bileaflet mitral valve prolapse and aortic root dilation (measuring 46 mm at the level of the sinuses of Valsalva). The patient has been prescribed metoprolol tartrate and losartan to control his aortic root disease and was to undergo elective aortic repair when the aortic dimension becomes near to or reaches 50 mm. A diagnosis of Marfan syndrome was made based on the revised Ghent criteria as the proband had aortic root dilation (*Z*-score: 3.72) and a systemic score of 12 (Table 1). His diagnosis was further confirmed after undergoing* FBN1 *sequencing and deletion/duplication analysis, which detected a novel pathogenic variant in gene* FBN1, *denoted by c.5872 T>A (p.Cys1958Ser) (Figure 1).

## 3. Discussion

Arterial aneurysms are most commonly caused by atherosclerotic disease, especially in elderly patients aged over 60. Other etiologies, such as connective tissue disorders, should be investigated in younger patients such as ours [5, 6]. Fibrillin-1 microfibrils, through interactions with elastin and other proteins, provide structure to elastic and nonelastic connective tissues [7, 8]. In addition to the architectural functions, Fibrillin-1 plays an important role in regulating TGF-*β* complexes in the extracellular matrix. TGF-*β* signaling controls various processes at the cellular level, such as cellular growth, differentiation, and apoptosis [9, 10]. When Fibrillin-1 is defective, it disrupts the normal architecture of connective tissues. Elastic fibers, found in tissues such as aortic media, become disorganized and fragmented resulting in the loss of elastic properties which causes the vessels to be less compliant and more prone to dilation and aneurysm formation [10, 11].

Pathogenic variants are identified in 72–93% of individuals fulfilling a clinical diagnosis of MFS based on Ghent nosology [12, 13]. The likelihood of detecting a pathogenic variant decreases for those individuals not meeting Ghent criteria [12]. Large intragenic deletions in the* FBN1* gene have been detected in approximately 2% of individuals with MFS who did not have a pathogenic variant identified by sequence analysis [14].

In our case a pathogenic variant has been identified in the* FBN1* gene. The C1958S variant has not been published as pathogenic or been reported as benign to our knowledge. The C1958S variant is a nonconservative amino acid substitution, which is likely to impact secondary protein structure as these residues differ in polarity, charge, and size. This substitution occurs at a position that is conserved across species and in silico analysis predicts this variant is probably damaging to the protein structure or function. Additionally, C1958S affects a Cysteine residue within a calcium-binding EGF-like domain of the FBN1 gene, which may affect disulfide bonding and is predicted to alter the structure and functions of the protein. Cysteine substitutions in the calcium-binding EGF-like domains represent the majority of pathogenic missense changes associated with FBN1-related disorders [2]. Furthermore, other missense variants at the same residue (C1958Y, C1958R) have also been reported in published literature in association with FBN1-related disorders, including assumed de novo occurrences for both variants, supporting the functional significance of this residue [15–17]. Finally, this variant has not been observed in large population cohorts such as the ExAC (Exome Aggregation Consortium) database [18–20]. Based on these data, ACMG variant classification guidelines [21] classify our variant as pathogenic (IIIa) (evidence: PM2, PS1, PM5, PP3, BS4, and PM1).

Peripheral vascular aneurysms in patients with Marfan syndrome have been reported on rare occasions [5, 22–24]. Bilateral popliteal aneurysms in MFS have been described on two occasions before [5, 23], but in one of these studies, it was believed that autosomal dominant polycystic kidney disease (ADPKD) was the main contributor to this manifestation [5]. In our case the patient did not have any other medical conditions besides his MFS diagnosis. Popliteal aneurysms should be properly investigated and repaired, as they can result in serious complications such as thrombus formation or rupture.

Based on history, the mother's mitral valve prolapse with severe regurgitation was attributed to rheumatic valve disease rather than a genetic connective tissue disorder. However, it is still a very likely possibility as her son has a confirmed diagnosis of MFS. She is yet to undergo the FBN1 sequencing.

Due to its rarity in MRS we do not recommend screening for peripheral vascular aneurysms in patients diagnosed with MRS as we do not think it is a cost-effective approach. However, we do recommend that patients with popliteal or other peripheral aneurysms, especially those who are relatively young and without risk factors, should undergo further clinical and genetic assessment in order to confirm/exclude the possibility of inherited connective tissue disorders.

In summary we describe the case of a 43-year-old male with a novel pathogenic variant in FBN1 causing Marfan syndrome with the rare phenotype of bilateral popliteal aneurysms. Further advancements in medical genetics will result in continued expansion and discovery of phenotypes as described above.

## Figures and Tables

**Table 1:** 

Feature	Value
Wrist and thumb sign	3
Pectus carinatum deformity	2
Hindfoot deformity	2
Plain flat foot	1
Scoliosis	1
Skin striae	1
Severe myopia	1
Mitral valve prolapse	1
Total	12

## Abbreviations

MFS: Marfan syndrome
ACL: Anterior cruciate ligament
ADPKD: Autosomal dominant polycystic kidney disease.
